# Cortical Neuroprosthesis Merges Visible and Invisible Light Without Impairing Native Sensory Function

**DOI:** 10.1523/ENEURO.0262-17.2017

**Published:** 2017-12-20

**Authors:** Eric E. Thomson, Ivan Zea, William Windham, Yohann Thenaisie, Cameron Walker, Jason Pedowitz, Wendy França, Ana L. Graneiro, Miguel A.L. Nicolelis

**Affiliations:** 1 Department of Neurobiology, Duke University, Durham, NC 27710; 2 Biomedical Engineering, Duke University, Durham, NC 27710; 3 Psychology and Neuroscience, Duke University, Durham, NC 27710; 4 Duke Center for Neuroengineering, Duke University, Durham, NC 27710; 5 Edmond and Lily Safra International Institute of Neuroscience of Natal, Natal, 59066-060, Brazil; 6 Department of Biology, École Normale Supérieure De Lyon, Lyon, 69342, France; 7 School of Medicine, University of California San Diego, La Jolla, CA 92093; 8 Florida International University, Miami, FL 33199

**Keywords:** Behavior, multisensory integration, neuroprosthesis, plasticity

## Abstract

Adult rats equipped with a sensory prosthesis, which transduced infrared (IR) signals into electrical signals delivered to somatosensory cortex (S1), took approximately 4 d to learn a four-choice IR discrimination task. Here, we show that when such IR signals are projected to the primary visual cortex (V1), rats that are pretrained in a visual-discrimination task typically learn the same IR discrimination task on their first day of training. However, without prior training on a visual discrimination task, the learning rates for S1- and V1-implanted animals converged, suggesting there is no intrinsic difference in learning rate between the two areas. We also discovered that animals were able to integrate IR information into the ongoing visual processing stream in V1, performing a visual-IR integration task in which they had to combine IR and visual information. Furthermore, when the IR prosthesis was implanted in S1, rats showed no impairment in their ability to use their whiskers to perform a tactile discrimination task. Instead, in some rats, this ability was actually enhanced. Cumulatively, these findings suggest that cortical sensory neuroprostheses can rapidly augment the representational scope of primary sensory areas, integrating novel sources of information into ongoing processing while incurring minimal loss of native function.

## Significance Statement

Using a sensory neuroprosthesis that projects information from the IR environment to primary sensory areas, we show that adult rats can rapidly integrate completely novel sensory information into preexisting cortical maps. When the prosthesis is implanted in V1, animals can learn to perform a multimodal integration task, fusing IR and visual information that is simultaneously superimposed on the same cortical area. When the prosthesis is implanted in S1, the tactile function of S1 is left undisturbed, and often enhanced. Hence, it is possible to merge multiple streams of information onto the same primary cortical area without compromising its original function. This is auspicious for the development of sensory prosthetic systems for adult victims of brain injury.

## Introduction

A fundamental goal in neuroscience is to delineate the mechanisms and limits of adult neurobehavioral plasticity. This task is important for both basic neuroscience and modern rehabilitative medicine (Lledo et al. 2006). This question was previously addressed using a real-time closed-loop sensory prosthetic system that allowed researchers to investigate how adult mammals responded when information from a completely new source, infrared light (IR), was projected to primary sensory regions (Thomson et al. 2013; Hartmann et al. 2016). In this cortical neuroprosthesis, the output of four head-mounted IR detectors was coupled to topographically distributed stimulating electrodes chronically implanted in the somatosensory cortex (S1) of adult rats (Thomson et al. 2013; Hartmann et al. 2016). As with ablation-induced sensory-cortex rewiring in newborns (Frost and Metin, 1985; Sur et al. 1988), adult rats readily learned to use this new source of information, ultimately performing as well as in corresponding visual discrimination tasks. Similar results were also observed when information from the geomagnetic environment was projected to V1 (Norimoto and Ikegaya, 2015).

Such initial results with cortical prosthetic systems raised several key questions that we presently address. One, because of the facility with which S1 absorbed information about IR sources, we postulated that different primary sensory areas should display similar levels of sensory plasticity. We advance this thesis based on previous results, but also on anatomic grounds: similar canonical microcircuits are iterated across the neocortical mantle (Haeusler and Maass, 2007; Kouh and Poggio, 2008; Habenschuss et al. 2013; Harris and Shepherd, 2015). To test this equipotentiality of plasticity hypothesis, we implanted stimulating electrodes in primary visual cortex (V1) of adult rats to measure their ability to use the IR neuroprosthesis and compare this with the performance of animals that used an S1-based prosthesis.

In the original rewiring experiments, performed in newborns, the transformed areas received inputs from only one sensory modality–for instance, rewired A1 received only visual inputs (Sur et al. 1988; von Melchner et al. 2000). In contrast, in animals using the IR prosthesis, both native and IR information was simultaneously available to the primary sensory area implanted with the prosthesis, and this allowed us to ask additional questions about how the brain handles the simultaneous superposition of multiple sources of information.

For instance, can the brain integrate both information sources simultaneously to perform a multisensory integration task? (Deneve and Pouget, 2004; Ghazanfar and Schroeder, 2006; Macaluso, 2006; Stein and Stanford, 2008; Klemen and Chambers, 2012). To examine this question, we developed a visual-IR multisensory integration task, in which animals were presented with multiple visible and IR cues simultaneously, and rats were rewarded only if they ignored distractor visual and IR cues, and selected the stimulus that combined both visible and IR lights. As far as we can tell, no previous sensory prosthetic system has been tested with a behavioral task with such a high degree of difficulty, requiring subjects to integrate information from two qualitatively different sources simultaneously superimposed onto the same cortical area.

Further, when a new source of information is projected to a cortical area, does this have unintended deleterious side effects? There are multiple lines of evidence that intracortical microstimulation (ICMS), by itself, is a powerful inducer of cortical plasticity (Recanzone et al. 1992; Maldonado and Gerstein, 1996; Godde et al. 2002). However, there is also evidence from monkeys that repeated microstimulation in V1 can impair performance on visual detection tasks (Ni and Maunsell, 2010). Also, there is evidence from the motor system of monkeys that ICMS can even highjack the cortex, displacing its original function (Griffin et al. 2011). To directly address the question of how a prosthetic system used in a discrimination task affects the original function of the implanted area, we examined how an S1-implanted prosthesis influenced performance on a whisker-based tactile discrimination task. The results will have potential clinical implications for neuroprosthetic design, as well as more basic implications about the limits of plasticity of a given region of primary sensory cortex.

## Materials and Methods

### General

All experiments were performed on adult female Long-Evans rats (Harlan Sprague Dawley Laboratories), 250–300 g. All animals were treated humanely to minimize stress, and the Duke University Institutional Animal Care & Use Committee (IACUC) approved all surgical and behavioral methods.

### Behavioral methods

#### Behavior chamber

**Figure 1.**
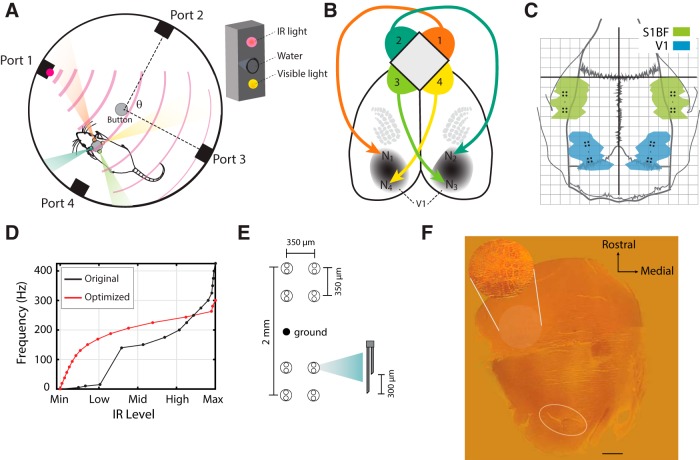
Methods. ***A***, Setup of the IR behavior chamber. Four reward ports line the walls of the circular chamber. The angle θ indicates how far apart adjacent ports are. Inset shows design of each individual port. ***B***, Illustration of the mapping from four IR detectors (1–4) to four microstimulating electrode locations (N_1_–N_4_) in V1. ***C***, Dorsal view of rat skull showing the placement of the S1 and V1 stimulating electrode arrays. The green and blue zones correspond to the S1 barrel field (S1BF) and V1, respectively, and these areas were reconstructed from (Paxinos and Watson, 2007). The dark horizontal and vertical reference lines intersect at bregma, and each small square in the grid is 1 mm^2^. ***D***, Transforms from IR levels detected in individual IR detectors to microstimulation frequency in individual stimulating channels. The black line is the original transform used in previous articles, and some animals in the present article. The red line shows the optimized transform (see Methods). ***E***, Design of electrode arrays consisting of two bundles of eight stimulating electrodes placed 2 mm apart (see Methods for more details). ***F***, Flattened cortical slice from animal implanted in V1, stained with cytochrome-oxidase. Inset shows detail of macrovibrissae barrels. Oval at bottom (caudal) shows the location of tissue damage from the V1 implant. Reference line: 1 mm.

The majority of behavioral experiments were performed in a cylindrical behavioral chamber (50.8 cm or 20 inches; Fig. 1*A*). A button was placed in the center of the chamber that allowed the rats to initiate a trial. In some sessions, the chamber included a mechanical button; because the mechanical button was difficult to learn to use, it was later replaced with an infrared photobeam recessed into the floor. Each chamber contained four ports around its circumference, initially evenly spaced 90˚ apart (Fig. 1*A*). On the inner surface of each port there was a visible LED and an infrared LED (Opto Semiconductors; 940-nm peak emittance with a range of non-zero emission between 825 and 1000 nm). The IR sources had an angular width at half-maximum of 120°. Each port contained a water spout inside a conical recess that also included an infrared photobeam, allowing us to detect when the rat selected that spout (Fig. 1*A*, inset).

#### Visual discrimination task

In the visual discrimination task, the animal initiated each trial by pressing a button in the center of the behavioral chamber, and then a single visible light was turned on in a single port. The animal received a small amount of water if it selected that port. Otherwise, an error tone sounded and there was a timeout delay (4–10 s) before the next trial could be initiated. We considered threshold performance to be when an animal reached 85% correct and performed at least 150 trials in a session, at which point the animal was ready to be implanted with stimulating electrodes and learn the IR discrimination task.

A total of 35 rats were trained on the visual discrimination task (21 to compare S1 and V1 learning rates on the basic IR discrimination task, 6 to examine visual-IR integration—3 in S1 and 3 in V1—and 8 to examine how whisker discrimination is influenced by microstimulation in S1).

#### IR discrimination

Once an animal reached threshold performance on the initial visual discrimination task, we trained them to discriminate single IR light sources in the same behavioral chamber. We first implanted an electrode array into the appropriate area (either S1 or V1, as described under Surgical procedures). After at least a week of healing from surgery, and after determining current thresholds for the four stimulating channels (see Microstimulation), we then trained them on a task that was structurally identical to the initial visual discrimination task. The only difference was that we replaced the visual light with IR light. Each rat was equipped with four IR sensors distributed evenly around the circumference of the animal’s head on the horizontal plane (Fig. 1*B*). The IR sensors (Lite-On) had a peak spectral sensitivity at a wavelength of 940 µm and a 20° width at half of its maximum sensitivity.

After processing (see IR→Stim transform), information from each sensor was projected to a stimulating channel at a different cortical location. The information was projected in a topographically natural way: for instance, information from the left sensors was projected to the right cortical hemispheres, and information from the anterior sensor was projected to the anterior region of S1, which represents the rostral whiskers (Hartmann et al. 2016). A similar pattern was replicated in V1 (Fig. 1*B*, *C*). Note also that surgeries to implant S1 and V1 were randomly interleaved, so as not to introduce bias or trends in the procedures.

During training on the IR-discrimination task, the visual lights were progressively replaced with IR lights. Specifically, they began with a mixture of two types of trials: IR only trials, in which only IR lights were turned on, and IR + vis trials, in which IR light (and ICMS) was shown, followed by visible light, to help animals associate ICMS with reinforced visual light. Specifically, on IR + vis trials, we would turn on the IR light, and then 400–500 ms later, turn on the visible light, and then 500 ms later, turn off the IR light for the rest of the trial. On their first day of training, the percentage of IR-only trials was 20%, and this increased to 100% by the fifth day of training. All percentage correct values on IR discrimination were based on IR-only trials.

A total of 41 rats were trained on the IR discrimination task (21 used to compare learning rates on the basic IR discrimination task, 6 to look at visual-IR integration, 8 to look at whisker discrimination, and 6 trained in the naive task; these 6 were not pretrained on the visual task). We did not perform *a priori* power calculations to determine the number of animals to use in the study.

#### Integrated IR + visual discrimination

In the integrated IR + visual discrimination task, we trained six animals to ignore visual and IR distractors and select the port in which both the IR and visual lights were activated simultaneously. On each trial, each of the four ports was randomly assigned one of four stimulus conditions: none, IR only, visual only, or both IR and visual (see Results). The IR- and visual-only ports were distractors, and the animal received a reward only when it approached the port with both IR and visual lights on.

#### Naive training

One group of six animals was taught to perform the IR discrimination task without being pretrained in the visual discrimination task. We first placed these naive rats in the behavioral chamber with only a single port placed along its circumference, to familiarize them with the behavioral chamber. Using the single-port setup, the rat was taught to poke a center button to initialize trials and subsequently poke the port to receive a reward. To avoid generating a directional bias in the rats, we randomly selected a position in the chamber’s circumference where the port would be placed every session. Once they were familiarized with the chamber (performing 100 trials per session for 3 d), we proceeded to implant them with stimulating electrodes in either S1 or V1. We then trained the rats using only infrared trials (no mixed visual + IR trials as in the standard IR discrimination protocol). The trainer was blind to the implant location: an assistant was in charge of setting a rat in the chamber while the trainer would make adjustments to the stimulation currents during the session. Every day, the order of the animals was randomized, and the software was written so that the trainer could only adjust currents relative to the initial current.

#### IR→Stim transform

The IR to microstimulation frequency transform is a step function from IR sensor voltage (which we sometimes refer to as “IR level” or “IR intensity”) to microstimulation frequency as shown in Fig. 1*D*. The original transform, described in Thomson et al. (2013), was roughly exponential in shape, with 17 step intervals. In the previous article, researchers used this transform to stimulate rats when neuronal activity was not simultaneously recorded. When online recording was introduced during the task to limit bandwidth, the transform was restricted to only seven intervals (Hartmann et al. 2016).

We used this same transform, but also developed a new optimized transform such that each stimulation frequency would occur with equal probability over the course of a session. To this end, we measured the probability of different voltage values occurring across multiple sessions. We then chose a new transform that would guarantee that each voltage step would occur with equal frequency, thereby increasing the effective information available to the animal on each trial. The voltage steps required to guarantee equal likelihood of occurrence were roughly logarithmically spaced, and we increased the frequency constantly for each step, so the transform from IR level to stimulation frequency is logarithmic, as seen in Fig. 1*C*. It is important to mention that we used all frequencies, and we observed no harmful effects [we previously have avoided frequencies 20–100 Hz (Thomson et al. 2013; Hartmann et al. 2016) for fear of inducing kindling (Goddard, 1967)].

#### Optimizing the sensitivity of IR sensors

IR sensors played a crucial role in the IR prosthetic system. The IR photodetector circuit included a simple voltage divider that let us adjust the sensitivity of the sensor. In practice, changing the resistance by 100 kΩ decreased the measured output of the IR sensor by ∼20% at a given IR level. This setup allowed us to scale the sensitivity in each IR sensor independently. In the IR-visual integration task, rats performed the task with their sensitivity set at a high value (resistance set to 475 kΩ) for several days until performance plateaued, usually 75% correct for 5 consecutive days. We then decreased sensitivity in steps, allowing the animal to reach a behavioral plateau at each new sensitivity level. For each rat, the optimum IR sensitivity was selected based on the best performance plateau, calculated over all sensitivity levels tested.

#### Aperture-width discrimination

We trained eight rats to discriminate the width of a tunnel using only their facial whiskers, a task discussed extensively previously (Krupa et al. 2001, 2004; Wiest et al. 2010). More specifically, we trained them to use their whiskers to discriminate the size of a variable-width aperture. Rats were trained to sample the width of a variable-width aperture using their whiskers (Fig. 4) and were rewarded for moving to the left reward port when the aperture was narrow and to the right reward port when the aperture was wide (see Results). Once they reached criterion performance (80% correct) with widths of 54 and 78 mm, we then switched some rats to a multiple width version (with either 12 or 14 widths). For the multiple width task, widths less than 66 mm counted as narrow.

### Surgical procedures

Surgeries were performed under ketamine (Ketaset) and xylazine (AnaSed, Akorn Animal Health) anesthetic with 100 mg/kg ketamine and 0.06 mg/kg xylazine. After cleaning the surface of the skull, we placed three to six titanium screws (Antrin Miniature Specialties) into the skull, sealed them in place with Metabond (Parkell), and coated the rest of the cleaned skull surface with Metabond before continuing with the surgery.

We then performed the craniotomy, removed the dura, and implanted electrodes that were built in-house (see Electrode design). The coordinates for S1 were –2.5 mm posterior and 5.5 mm lateral to bregma. The V1 coordinates were –7.45 posterior and 3.25 lateral to bregma, oriented 30.5° relative to the midline (with the anterior portion more lateral; Fig. 1*C*). After the electrodes were lowered to the proper depth (1.5 mm in S1, and either 0.8 or 1.3 mm in V1; Results), we sealed the craniotomy with Quick Set dental acrylic (Coltene) and cyanoacrylic (Hobbylinc.com). Locations of electrodes were verified histologically (see below) and physiologically by examining manual responses to whisker deflections (S1) and LED flashes (V1).

### Electrophysiology

#### Electrode design

The stimulating/recording arrays consisted of 32 42-µm stainless steel microwires arranged into four groups of eight (see Fig. 1*E*; Hartmann et al. 2016). Microwires were paired, such that each cortical penetration contained two microwires, with the potential for each couple to be used as an anodic/cathodic dyad for microstimulation (see Microstimulation). The pairs were 300 µm apart, and this was also the distance between different adjacent wires in the same group. Clusters were 2 mm apart within a hemisphere (Fig. 1*E*). All electrodes were designed and built in-house.

#### Histology

For histologic verification, we used cytochrome oxidase (CO) staining of flattened cortical sections, as described extensively elsewhere (Wong-Riley, 1979; Hartmann et al. 2016). Briefly, after perfusing the animal with 0.1 m phosphate buffer, we removed subcortical tissue and flattened the cortex overnight in 30% sucrose. After freezing, we sliced the brain at 40–60 µm and collected free-sections in 0.1 m phosphate buffer (pH 7.4) and placed them in standard CO reaction solution (Wong-Riley, 1979). We gently agitated the slices at room temperature for 2–6 h until the sections appeared golden-brown to the eye (Fig. 1*F*).

#### Recording

Single- and multiunit extracellular neurophysiological data were recorded using the Multichannel Acquisition Processor (MAP; Plexon). After collecting data, during offline sorting we selected spike wave form features (principal components, peak amplitude, etc.) that provided optimal separation using Offline Sorter software (Plexon; typically we used the principal components, but sometimes we used other features such as wave form peak or wave form energy). Single-unit quality was determined by separation of the unit from the noise in the 2- or 3-D feature space and the presence of a clear absolute and relative refractory period in the autocorrelogram for the putative unit.

#### Microstimulation

We stimulated using a four-channel biphasic, charge-balanced microstimulator (Hanson et al. 2012). Each stimulation pulse consisted of a 100-µs pulse from the anodic wire, followed by a 50-µs pause, followed by a 100-µs pulse in the cathodic wire.

After the animals recovered from surgery, we determined the minimal current threshold for four stimulating channels, one in each location. In awake relaxed animals, we would deliver a 250-Hz train of stimulation for 75 ms, starting with a low current (5 µA), and incrementally increased the current amplitude until we visually saw a reaction from the animal (a movement of the head, locomotion, etc.). We also measured neuronal thresholds: we applied 250-Hz trains of stimulation for 75 ms in isoflurane-anesthetized animals and noted the current amplitude at which we observed a response above baseline (the threshold current), and also the amplitude at which the neuronal responses saturated (the saturating current, which is the current above which we did not observe an increase in response).

In practice, we have found that animals learn the task faster when we apply currents well above the threshold for evoking a response, as long as the currents are not aversive to the animal (Hartmann et al. 2016). Hence, for our initial currents for the prosthesis, we typically started well above the neuronal threshold, at twice the saturating current (typically 50–90 µA). We took it as a sign that a current was aversive if the animal began scratching its face or stopped participating in the task in response to ICMS.

#### Whisker receptive field mapping

To determine the principal whiskers that evoked activity in an S1 unit, we recorded in lightly anesthetized (isoflurane) animals while stimulating individual whiskers with a hand-held probe or air puff and logged the whiskers that evoked a noticeable change in the baseline response on an audio monitor. Note that the goal was to quickly determine which whiskers needed to be trimmed during the whisker discrimination task, not to construct detailed quantitative receptive field maps, so we used this relatively coarse method to minimize the animals’ time under general anesthesia.

### Analysis

#### Stimulus population vector

To compactly represent the set of four microstimulation bursts delivered to the brain at a given time, we employed a stimulus population vector, which is based on population vector representations of movement from the motor control literature (Georgopoulos et al. 1986). Briefly, it provides an intuitive geometric 2-D representation of the full stimulus being delivered to the four cortical locations at a given time. For instance, if the two anterior stimulating channels are maximally activated, then the population vector points to the front. If the two left channels are active, it will point to the left, etc. Mathematically the stimulus population vector at time *t*, **s**(*t*) is defined as

$$s\left(t\right)=\overset{4}{\underset{i=1}{\sum }}f_{i}\left(t\right)v_{i},$$

where *f_i_*(*t*) is the microstimulation frequency in channel *i*, and ***v****_i_* is a vector that points in the direction of IR channel i on the rat’s head (for instance, ***v***_1_ = <1,1>: see Fig. 1*B*). For more discussion of the stimulus population vector, see Results and Fig. 3. For a more extensive introduction, see (Hartmann et al. 2016).

#### Psychometric curve fitting

For the aperture-width discrimination task in which 12 or 14 widths were presented, we fit the behavioral data to a Weibull function: a saturating exponential function with four free parameters. We used Matlab to fit our behavioral data, in the least squares sense, to the following:

$$\min+\left(\max-\min\right)\left[1-e^{-{\left(\frac{x}{\lambda }\right)}^{k}}\right],$$

where min is the minimum value, max is the maximum value, *k* is the shape parameter, and λ is the scale parameter. This is the standard Weibull curve modified to be constrained to have a minimum and maximum value (it typically varies between zero and one).

#### Statistics

The different effect size calculations for Table 1 depend on the statistic used as described in detail in Cohen (1988) (Thompson, 2006; Faul et al. 2007; Olivier and Bell, 2013). For *t* tests, we used Cohen’s *d* (Cohen, 1988; Faul et al. 2007). For ANOVA measures, we used Cohen’s *f*, first calculating η^2^ as the ratio of variance explained SS_effect_/SS_total_ (Thompson, 2006) and then using the standard conversion to *f* (Cohen, 1988; Thompson, 2006):

$$f=\sqrt{\frac{\eta^{2}}{1-\eta^{2}}.}$$

**Table 1.** Statistical analysis

Figure	Test	Quantities compared	*P*-value	Effect size
2*B*	Two-tailed *t* test	S1 vs. V1: num sessions to learn	0.0032	1.6259
2*E*	Two-tailed *t* test	S1 vs. V1: num trials to learn	0.0047	1.6048
2*F*	ANOVA	V1: percent correct (PC) vs. difficulty	0.0015	0.9904
2*F*	Two-way ANOVA	Factor 1 (angle): mean PC	0.0003	0.4261
2*F*	Two-way ANOVA	Factor 2 (implant location): mean PC	0.6186	0.043
2*F*	Two-way ANOVA	Interaction (angle × location): mean PC	0.5024	0.1336
2*G*	Two-tailed *t* test	Naive V1 vs. pretrained S1: num sessions to learn	0.8518	0.1201
2*G*	Two-tailed *t* test	Naive S1 vs. V1: num sessions to learn	0.6433	0.4082
3*E*	*F* test	Stimulus location vs. receptive field peak correlation	0.7	0.0259
4*C*	Two-tailed *t* test	S1 vs. V1: num sessions to learn integrated task	1	0
4*D*	Two-tailed *t* test	S1 vs. V1: num trials to learn integrated task	0.6258	0.4306
5*E*	Two-way ANOVA	Factor 1 (animal group): PC for two-width case (54/78)	0.5485	0.0833
5*E*	Two-way ANOVA	Factor 2 (treatment): PC for two-width case	0.0004	1.115
5*E*	Two-way ANOVA	Interaction (group × treatment): PC two widths	0.8597	0.4738
5*E*	Two-tailed *t* test	Two-width PC change versus zero: after stimulation	0.0136	1.1567
5*E*	Two-tailed *t* test	Two-width PC change versus zero: after clipping	0.0014	2.0877
5*F*	Two-way ANOVA	Factor 1 (animal group): PC multiwidth (12/14 widths)	0.9187	0.026
5*F*	Two-way ANOVA	Factor 2 (treatment): PC multiwidth	0.0179	1.1543
5*F*	Two-way ANOVA	Interaction (group × treatment): PC multiwidth	0.6384	0.1217
5*F*	Two-tailed *t* test	Multiwidth PC change versus zero: after stimulation	0.153	0.6874
5*F*	Two-tailed *t* test	Multiwidth PC change versus zero: after clipping	0.009	2.1161
5*G*	Two-way ANOVA	Factor 1 (animal group): sensitivity	0.7111	0.0833
5*G*	Two-way ANOVA	Factor 2 (treatment): sensitivity	0.0105	1.115
5*G*	Two-way ANOVA	Interaction (group × treatment): sensitivity	0.0868	0.4738
5*G*	Two-tailed *t* test	Sensitivity change versus zero: group after stim	0.0107	1.6174
5*G*	Two-tailed *t* test	Sensitivity change versus zero: group after clip	0.3449	0.4786
6*E*	Chi-squared test	Proportion w/anticipatory response in stim vs. control	0.009	0.18
6*E*	Chi-squared test	Proportion w/response to stim in stim vs. control	0.053	0.13

Similarly, for regression analysis (which used an *F* test for significance), we defined the effect using the same measure, but with the regression coefficient *r* taking the place of η (Cohen, 1992; Faul et al. 2007). For chi-squared tests, we used Cohen’s ω statistic (Cohen, 1992; Faul et al. 2007; Olivier and Bell, 2013):

$$\omega =\overset{K}{\underset{i=1}{\sum }}\frac{{\left(p_{oi}-p_{ei}\right)}^{2}}{p_{ei}},$$

where *p_ei_* is the expected frequency of observations, under the null hypothesis, to fall in group *i*, and *p_oi_* is the observed frequency in group *i*. All calculations of effect size were conducted in Matlab.

## Results

To examine the ability of primary visual cortex (V1) to absorb a new and otherwise invisible source of electromagnetic signals, we used ICMS to deliver information generated by IR sources directly to V1. Rats first learned to discriminate visual cues in a circular behavioral chamber with four light sources (see Methods; Fig. 1*A*). We then implanted them with an IR prosthesis and trained them on an IR discrimination task in which they had to correctly identify which of four ports in the behavioral chamber had an active IR source (Fig. 1*A*).

Infrared information was coupled to V1 via four IR sensors that were arranged around the circumference of the rat’s head, so they were able to perceive a full 360° view of the circular arena (see Methods; Fig. 1*B*). We implanted stimulating electrodes bilaterally in V1 and projected information from IR sensors to the cortex in a topographically natural manner, such that the information from the left IR environment was projected to the V1 of the right hemisphere (see Methods; Fig. 1*B*; Hartmann et al. 2016).

**Figure 2.**
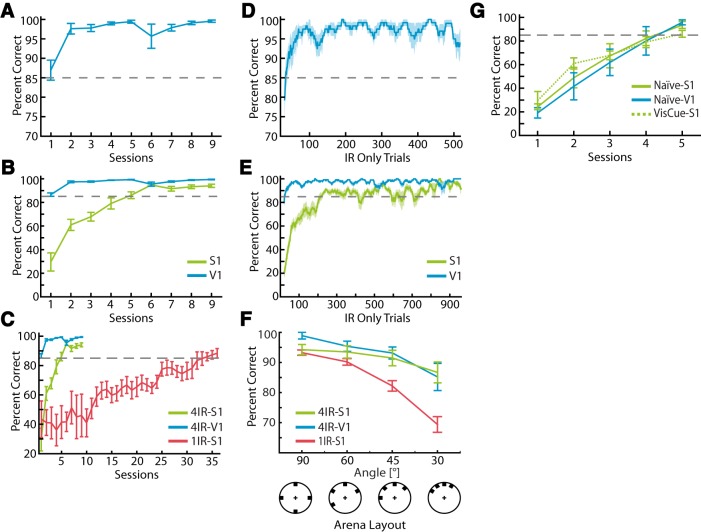
Discriminating IR light with visual cortex. ***A***, Learning curve for IR-discrimination task using V1 (*n* = 6 animals). Note animals typically surpassed criterion (85% correct) within the first day. ***B***, Direct comparison of learning curve for the same task with V1 implants (blue) and S1 implants (green; *n* = 15 for S1 implants). ***C***, Same information as in ***B***, but with learning curve superimposed when an animal has just a single IR sensor on its head (single-sensor learning data are from a previous study [Thomson et al. 2013]; *n* = 4 animals). ***D***, Trial-based moving average analysis of performance in IR discrimination task. The curve shows percentage correct as a function of trial number (moving average of 20 trials) from same data shown in ***A***. ***E***, Same analysis as in ***C***, but with S1-implanted animals shown in green for direct comparison. ***F***, Performance in IR-discrimination task as a function of angle θ between the ports (see Fig. 1*A*). Includes data from V1 and S1 implanted animals with four IR sensors, as well as data from S1-implanted animals with a single IR sensor for comparison (blue, green, and red lines respectively with mean ± SEM percentage correct). ***G***, Performance of naive V1- and S1-implanted animals (mean ± SEM percentage correct), that were not pretrained on a visual discrimination task (solid lines). Data from S1-implanted animals that were pretrained on a visual discrimination task are included for comparison. There is no significant difference, among any of the three groups, in the number of sessions it takes to reach 85% correct in the task (*p* > 0.05, two-tailed *t* test).

Surprisingly, when IR information was delivered to V1, rats learned to discriminate IR sources extremely quickly, often within their first day of training (Fig. 2*A*). This was the case in four of six rats. Quantitatively, the V1-implanted animals surpassed 85% correct in the IR discrimination task in 1.3 ± 0.2 d of training (*n* = 6 animals). This is significantly faster than S1-implanted animals, who took 3.8 ± 0.4 d to surpass 85% correct (*n* = 15 rats; *p* = 0.003; two-tailed *t* test; Fig. 2*B*). Note that we have significantly more S1-implanted animals because we were able to include data from eight animals from a previous study (Hartmann et al. 2016). For comparison, Fig. 2*C* shows the learning curve when information from a single IR sensor was projected to S1 (data are from a previous study, Thomson et al. 2013). In this case, animals took on average 22.3 ± 7.6 d to reach 85% correct on the task.

Because the V1-implanted animals were clearly learning to perform the IR-discrimination task within the first session, we examined how many trials it took them to learn the task, using a 20-trial moving average (Fig. 2*D*). When IR information was delivered to V1, it took them on average 26.0 ± 4.0 IR-only trials to reach 85% correct, versus 138.6 ± 21.1 IR-only trials when projected to S1 (Fig. 2*E*), a significant difference (*p* = 0.0047, two-tailed *t* test). During training, such IR-only trials were interleaved with mixed trials in which IR light was followed by visible light, to facilitate learning the association between IR light and reward (see Methods). The total number of trials (both IR-only and mixed) required to learn the task was 174.33 ± 22 for V1-implanted animals and 600.2 ± 67 for S1-implanted animals, a significant difference (*p* < 0.001; two-tailed *t* test).

To examine the spatial acuity of the animals’ ability to discriminate IR lights, once they were above criterion at the initial task, we varied the angle θ between the ports (Fig. 1*A*). When the ports are placed closer together (i.e., θ is decreased), task difficulty is increased because multiple IR lights can activate the same IR sensor, thereby generating ambiguity in the stimulus. We found that performance in the task decreased as ports were moved together from 90˚ down to 30˚, which is the closest we could physically move the ports together in our setup (Fig. 2*F*). The decrease was small but statistically significant (*p* = 0.0015; ANOVA). Their performance as a function of angle was not significantly different from when implants were in S1 (*p* = 0.619; two-way ANOVA).

As a control, we then tested whether V1-stimulated animals learned to discriminate IR sources faster because they were pretrained in a visual discrimination task that was structurally identical to the subsequent IR discrimination task. That is, did the V1 animals learn faster because they were already using V1 for a visual task, so their learning generalized more quickly when microstimulation was applied to V1 (perhaps because they were already attending to V1)? To test this, we trained six animals on the IR discrimination task that had no pretraining on a visual discrimination task, three implanted in V1 and three in S1. The trainer was blind to the location of implant (see Methods). The hypothesis was that if the V1-implanted animals learned faster because of visual pretraining, then when they did not undergo such training, their performance should converge on that observed in the S1-stimulated animals. As can be seen in Fig. 2*G*, this is exactly what we observed. The naive V1-implanted animals took 4.0 ± 0.57 d to learn the IR discrimination task, which was not significantly different from the S1-implanted animals pretrained on a visual task, who took 3.8 ± 0.4 d (*p* = 0.85; two-tailed *t* test). The naive S1-implanted animals took 4.33 ± 0.33 d to learn the task, which was also not significantly different from the naive V1 animals (*p* = 0.64, two-tailed *t* test).

### Response to microstimulation in V1

To help understand how the brain is processing this new source of sensory information, we recorded from V1 neurons in animals that performed well above criterion at the task (16 sessions in four animals), using methods discussed extensively elsewhere (Hartmann et al. 2016). Recording during the task required that we change the stimulation protocol. Namely, instead of continuously updating the stimulating frequency every 50 ms, which produces continuous stimulus artifact, during recording we would stimulate the brain intermittently. That is, every 140 ms we would sample the IR levels, and stimulate for just 75 ms based on those levels, and then for the rest of that 140-ms period we would turn off the microstimulators, to allow for an artifact-free epoch for recording the neuronal response during that stimulus cycle.

**Figure 3.**
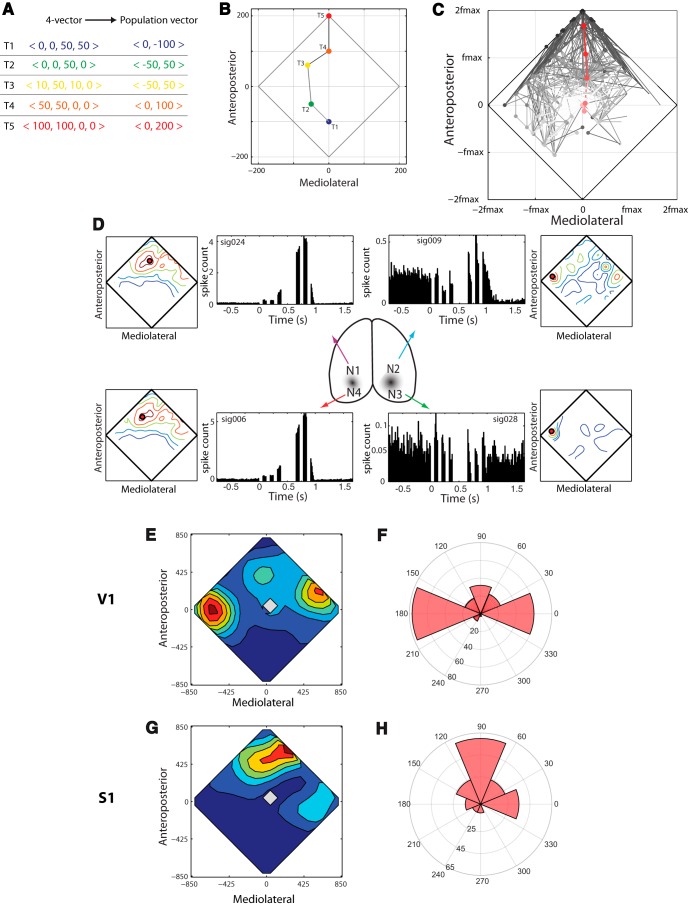
Neuronal responses to intracortical microstimulation in V1. ***A***, Example of reduction of four-element full representation of electrical stimulation vector (four-element vector where each number represents frequency of microstimulation at a different location) down to two-dimensional population vector. There are five examples from five different time points T1–T5. ***B***, Geometric representation of the population vectors from ***A***. ***C***, Stimulus population vectors from all trials in a session, with mean of first and last three substimuli overlaid in red. The black diamond outline is the convex hull of stimulus population vectors, showing the perimeter of the set of all possible vectors. Note that only a fraction of this set is actually presented in a given behavioral session. See text for more details. ***D***, Neuronal data from four representative units. PSTHs show mean response to first three and last three microstimulation pulses, averaged over all trials in the session (note there are different axis limits for the different PSTHs). Associated with each PSTH is an IR-RF, a contour plot that shows the mean number of spikes in response to each stimulus population vector, with hot colors representing high firing rates. ***E***, Distribution of all 240 IR-RF centers from all units recorded: note they tend to concentrate at the two medial corners of the stimulus space, although these are fairly rare events (***C***). ***F***, Polar count histogram illustrates the medial, rather than anterior, concentration of IR-RF angular distribution. ***G***, ***H***, Same as ***E*** and ***F***, but from data from S1 from a previous study (Hartmann et al. 2016). Note the anterior distribution of the IR-RF centers, much closer to the distribution of the stimuli.

To visualize the neuronal response to the distributed IR stimulus, we compactly represented the IR-based microstimulation patterns using a stimulus population vector [see Methods; Fig. 3*A*, *B* shows how such vectors are constructed; see also Hartmann et al. (2016)]. This vector provides a compact geometric 2-D representation of the full set of four 75-ms ICMS bursts delivered to the cortex at a given time. For instance, if the two anterior stimulating channels are maximally activated, then the population vector points to the front. If the two left channels are active, it will point to the left, and so on.

Fig. 3*C* shows the set of all population vectors delivered during one session in a V1-implanted rat. To show how the stimulus changed over time, contiguous stimuli within a trial are connected by a line, and later stimuli are indicated by darker circles in the figure. In a typical trial, the stimulating population vector started near the origin at the center of the graph, meaning that the microstimulating electrodes have very low levels of activation, and moved to the top (meaning that the two anterior microstimulating electrodes were activated). The mean of the first and last three stimulus population vectors are overlaid in red, showing that this trend of starting near the origin and moving anterior is representative.

We used peristimulus time histograms (PSTHs) to quantify the mean responses of V1 neurons to ICMS during the IR-discrimination task. Four representative PSTHs depicting the response of V1 neurons to microstimulation during the IR task are shown in Fig. 3*D*. These PSTHs display the mean neuronal response to the first and last three microstimulation bursts in a trial, averaged over all trials in a session. Note that during the time of microstimulation, the PSTH is zero because of ICMS-induced stimulus artifact, so each PSTH shows responses to microstimulation after this brief artifact period. There is a gap between the first and last three ICMS bursts to indicate that there were a variable number of substimuli on each trial.

Although they are useful portraits, such PSTHs provide a limited description of the full range of neuronal responses to ICMS as animals perform the IR discrimination task: the first and last three stimuli are incomplete samples of the full space of stimuli delivered to the brain during the task. To give a more complete picture of neuronal response, we used the IR receptive field (IR-RF), which measures the mean spike count as a function of stimulus population vector, with the count calculated for all population vectors delivered during a session. Fig. 3*D* shows the IR-RFs for four neurons, adjacent to their PSTHs. For instance, unit 28 preferred IR stimuli that were presented to the left of the animal. This explains why its firing responses to the first and last three stimuli, shown in the PSTH, were relatively small.

This lateralization of IR-RF centers was typical in V1, as can be seen in Fig. 3*E*, which shows a contour plot depicting the distribution of IR-RF peaks for all 240 V1 units recorded in this study. There was a pronounced trend for these V1 IR-RF peaks to be lateralized to the left and right sides. This is more clearly demonstrated by the polar count histogram of receptive field peak angular location in Fig. 3*F*. This is in sharp contrast to what was previously observed in S1, in which the IR-RF centers tended to closely match the stimulus statistics: that is, the S1 neurons had a strong preference for stimuli in the anteromedial sector of the stimulus space, as shown in Figs. 3*G* and 3*H*, which are reproduced from previous data (Hartmann et al. 2016).

Although previous research demonstrated the existence of a highly significant 2D correlation between IR-RF peak location and stimulus location in S1 (Hartmann et al. 2016), in V1 there was no such match between IR-RF peak and stimulus location: the 2-D correlation between IR stimulus distribution (the set of all stimulus population vectors) and IR-RF center was weak and not significant (ρ = –0.03; *p* = 0.7; *F* test).

### Integration of visual and IR information in V1

**Figure 4.**
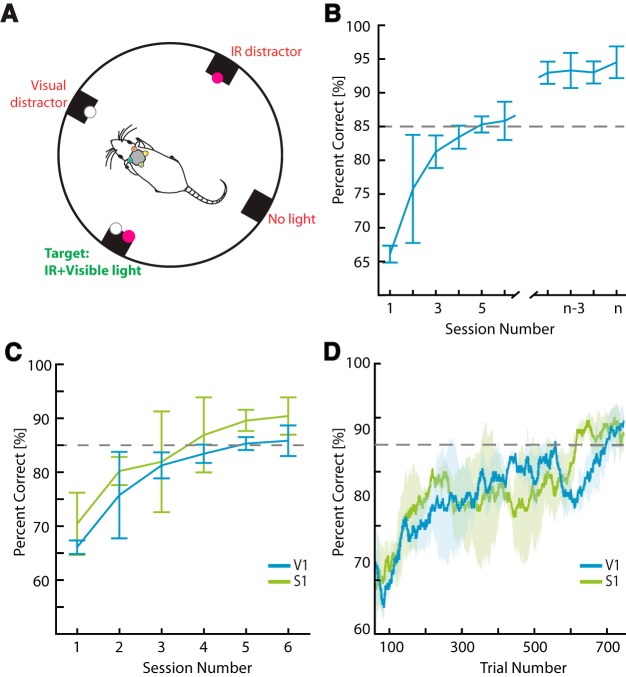
Visual-IR integration task. ***A***, Schematic of the task. In a cylindrical chamber with the same ports shown in Fig. 1*A*, four stimuli are randomly assigned to the ports. The target stimulus is the one with both visual and IR lights on. The IR distractor has only an IR light on, the visual distractor only a visual light, and there is one port with no light activated. ***B***, Learning curve from three animals trained on the visual-IR integration task (implants in V1). The discontinuous right portion of the curve shows the last four sessions using an optimized frequency (see text). ***C***, Comparison of learning curves between V1- and S1-implanted rats in the visual-IR integration task (*n* = 3 animals for each group). ***D***, Same data as in ***C***, but with trial-based moving average (moving window average taken with 20 trials).

When using the IR prosthesis, both native (visual) and novel (IR) information streams were projected to the same primary sensory area. To determine whether rats could learn to simultaneously integrate visual and IR information within V1, we trained them on a visual-IR integration task. During this task, we presented multiple visual and IR lights in the behavior chamber simultaneously. To receive a reward, rats had to select the single port in which both visible and IR lights were active (see Methods). On each trial, four stimuli were presented in the standard cylindrical behavioral chamber (Fig. 4*A*): in the target port, both the visual and IR lights were activated simultaneously. In another, a visual distractor light was turned on, while a third port contained an IR distractor light. The fourth port showed neither light. The location of each of these ports was selected randomly on each trial.

In preliminary testing, one cohort of three animals had trouble learning this task with high accuracy, often going to the first port that they noticed with an IR light activated. This seemed to be partly because they were being overstimulated in the relatively small chamber in which they were initially trained. To test this hypothesis, we trained a second cohort of three animals in a larger-diameter behavior chamber (20-inch or ∼0.5-m diameter), and significantly decreased the sensitivity of the IR sensors, so when a trial was initiated in the middle of the chamber, the IR sensors were not as likely to trigger ICMS at the start of the trial. Further, we optimized the transform from IR sensor output to ICMS frequency so that no frequencies were oversampled (see Methods; Fig. 1*D*).

After such optimizations were in place, animals readily learned to perform the visual-IR integration task, reaching 85% correct within 3.67 ± 0.67 sessions (*n* = 3 animals; Fig. 4*B*). Despite this performance, we noticed that some of the animals tended to make more errors toward the IR or visual distractor and hypothesized that they might be biased by microstimulation frequency. Hence, we systematically varied the maximum frequency of stimulation until we found the frequency that optimized percentage correct in the task (see Methods). Before frequency optimization, the best performance over five sessions was 82.7 ± 1.0%, and after optimizing frequency, the best performance over five sessions was 93.5 ± 1.4% (Fig. 4*C*). During initial training, we stimulated at a maximum frequency of 300 Hz and found that the optimal frequency, that minimized errors, was 133 ± 33 Hz.

We next tested the hypothesis that processing both IR and visual inputs in V1 would interfere with performance of the visual-IR integration task. In particular, we implanted a group of three animals with stimulating electrodes in S1 and trained them in the visual-IR integration task. In these animals, the IR information would be processed in S1, while the visual information would go to V1, so direct sensory interference between the two types of information would not occur. Yet, animals would still have to directly compare both sources of sensory information—IR light coming through S1 and visible light through V1—to solve the task properly. Hence, if the S1-implanted animals performed better on the visual-IR integration task, this would provide an indirect measure of sensory interference within V1 during the sensory integration task, while any residual errors could be interpreted as a measure of intrinsic task difficulty.

Surprisingly, the S1-implanted animals reached 85% correct within 3.67 ± 0.88 sessions (Fig. 4*C*), which was not significantly different from the V1-implanted animals (*p* > 0.5, two-tailed *t* test). When we analyzed the results by trial, we saw similar results (Fig. 4*D*). The S1-implanted animals reached 85% correct within 139 ± 51 trials, and the V1 implanted animals reached 85% correct within 103 ± 45 trials, and these learning rates were not statistically different (*p* = 0.626; two-tailed *t* test). These results suggest that ICMS in V1 does not appreciably impair the ability of V1 to process incoming visual stimuli while simultaneously receiving native visual information. Moreover, it demonstrates that rats can combine invisible (IR) and visible light, delivered to two different primary cortical areas (S1 and V1), as efficiently as when both sensory signals converge in their V1, to solve this difficult sensory integration task.

### Effect of prosthesis use on native tactile processing

**Figure 5.**
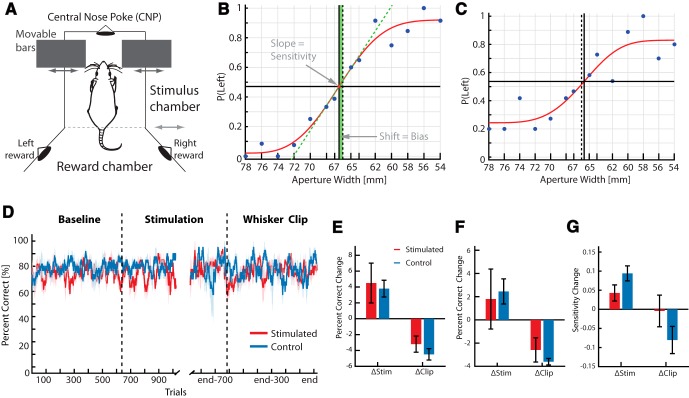
Effects of S1 prosthesis on whisker discrimination. ***A***, Aperture-width discrimination task. After the movable bars making up aperture move to the desired position, the rat enters the stimulus chamber, sweeps its whiskers across the aperture (movable bars controlled by the computer), and activates the central nose poke (CNP). The animal then retreats into the stimulus chamber and pokes in the right or left reward port depending on the aperture width. ***B***, Psychometric curve from a single session in the 14-width aperture-width discrimination task. The points are the mean proportion of left responses at each width; the red line is the best minimum least-squares fit to the points, fitted using a Weibull function. ***C***, As in ***B***, but in an animal with worse performance (lower maximum slope of the curve and lower peak performance at each end of the spectrum). ***D***, Performance of stimulated and matched control animal in the whisker discrimination task during different stages of the experiment. Before stimulation (Baseline), while learning the IR discrimination task via ICMS (Stimulation), and after whiskers were clipped (Clipped). See text for details. ***E***, Mean ± SEM change in performance after the two transitions illustrated in ***D***. ΔStim, transition from baseline to stimulation; ΔClip, transition from normal stimulation to stimulation with whiskers clipped. This analysis is restricted only to 54/78-mm widths, and includes eight animals (four stimulated/four control for the Δstim transition) and seven animals (four stimulated/three control) for the Δclip transition (one control animal stopped performing the task after whisker clipping). ***F***, Same as ***E***, but for the full multiple-aperture with discrimination task (either 12 or 14 widths). This includes six animals for the first transition and five animals for the second (as before, one control animal stopped behaving after whisker clipping). Percentage correct is calculated as the integral under the best Weibull fit to the psychometric curve (see Methods). ***G***, Change in behavioral sensitivity, or the measure of the maximum slope of the best fit curves to the psychometric data, with each transition in the task. Lower slope means less behavioral sensitivity.

In a final set of experiments, we further explored the consequences, for the native sensory modality, of projecting new information to a primary sensory area. Previous research has suggested that sensory prosthetic systems that employ ICMS might compromise the original function of the cortical region to which information is projected (Ni and Maunsell, 2010). Here, we measured whether rats in which IR information was projected to the whisker representation in S1 showed any decline in their ability to perform a whisker-dependent tactile discrimination task. Toward this end, we trained animals in two behavioral tasks each day: the stimulation-based IR discrimination task and a whisker-dependent aperture-width discrimination task (see Methods; Fig. 5*A*; Krupa et al. 2001, 2004; Thomson et al. 2014).

Briefly, in the aperture-width discrimination task, a variable-width aperture is moved to one of two widths (54 or 78 mm), and after sampling the aperture with its large facial whiskers, the rat must select the correct associated reward port to receive reward (Fig. 5*A*). We trained eight rats on this two-width discrimination task, and trained six of these rats on a multiwidth version of the task in which we randomly selected from more than just two widths (either 12 or 14). Widths >66 mm were considered wide, and those narrower than 66 mm were considered narrow. This allowed us to measure the bias (preference for one width over another), sensitivity (maximum slope of the psychometric curve), and standard overall percentage correct on the task over the full range of widths presented (Fig. 5*B*, *C*).

Once rats were trained on the two tasks, we implanted them with stimulating electrodes in S1 as before. We trained four rats to perform stimulation-based IR discrimination (the stimulated group) and four to perform the equivalent visual discrimination task as described above (the control group). This allowed us to track tactile discrimination performance in the two groups, each performing two similar tasks a day, with only one group receiving microstimulation in S1.

Fig. 5*D* shows the raw performance (percentage correct) in the multiwidth discrimination task in three key phases, in both the stimulated and control groups. The first, baseline, phase shows the percentage correct in both groups on the initial four-choice visual discrimination task, before any S1 microstimulation. The second, stimulation, phase shows performance on the aperture-width discrimination task after the stimulated group began training on the IR discrimination task. The third and final whisker clip phase was when all whiskers, except those corresponding to the barrels being stimulated, were clipped on each side of the face. It is important to note that at each phase, each rat was performing two tasks each day: the whisker discrimination task and either the IR discrimination task (stimulated group) or visual discrimination task (control group). Fig. 5*D* plots their performance *only* in the aperture-width discrimination task.

The third, whisker clipping, phase was crucial because it is known that rats can perform the aperture-width discrimination task above chance with just one whisker remaining on each side of the face (Krupa et al. 2001). Hence, to control for the possibility that the stimulated group could perform the task using the whiskers that were outside of the region being stimulated in S1, we clipped all of the facial whiskers that did not correspond to barrels that were stimulated by the prosthesis (stimulated group) or simply whiskers outside of the region of the implant (control group). After determining the whiskers that evoked activity at the site of S1 stimulation, we clipped the remaining whiskers on the face of the rat. This meant we clipped ∼20 whiskers, leaving only eight whiskers on each side of the face.

To determine whether ICMS, or trimming whiskers, altered the performance of the stimulated relative to control animals in the aperture-width discrimination task, we performed a 2 × 2 ANOVA [factor 1: animal group (control/stimulated); factor 2: treatment (stimulation/whisker clipping)]. Crucially, there was no significant difference in performance change between the two groups of animals using any of the measures, but there was a significant effect of treatment using each measure (α = 0.05: Figs. 5*E–G*; Table 1).

For instance, Fig. 5*E* shows the mean response change for all animals in the 54 vs. 78 whisker discrimination task, both when they were switched from the baseline to the stimulation phase (*Δ*Stim) and when they were switched from the stimulation phase to the whisker clip phase *(Δ*Clip). Fig. 5*F* shows a similar plot, but for percentage correct calculated using data from the multiwidth version of the task. Fig. 5*G* examines changes in sensitivity (the slope of the psychometric curve). Using all of these measures, there was no significant difference between groups. This suggests that microstimulation did not appreciably impair the ability of the stimulated rats to use their whiskers in the aperture-width discrimination task.

However, there were significant effects of treatment in each case (Table 1). Because the changes were similar in the two groups between phases of the task, to examine the sources of the differences we lumped the data from the stimulated and control groups together and looked for potential effects of each change in condition in a *post hoc* analysis. Surprisingly, percentage correct actually increased between baseline and stimulation phases, suggesting an effect of continued learning (Fig. 5*E*, *F*). This was significant in the eight animals trained on the two-width task (*p* = 0.01; two-tailed *t* test), but not significant in the multiwidth task (*p* = 0.153). Not surprisingly, the performance drop was significant after whisker clipping (*p* = 0.001 for the 54/78 task; *p* = 0.009 for the multiwidth task; two-tailed *t* test). The animals’ sensitivity increased during the stimulation period (a significant change: *p* = 0.01; two-tailed *t* test), and dropped after whisker clipping, although this drop was not significant (*p* = 0.34). Surprisingly, using all of these measures, the performance drop after whisker clipping was actually less for the stimulated group (Fig. 5*E–G*), but this trend was not significant (α = 0.05).

**Figure 6.**
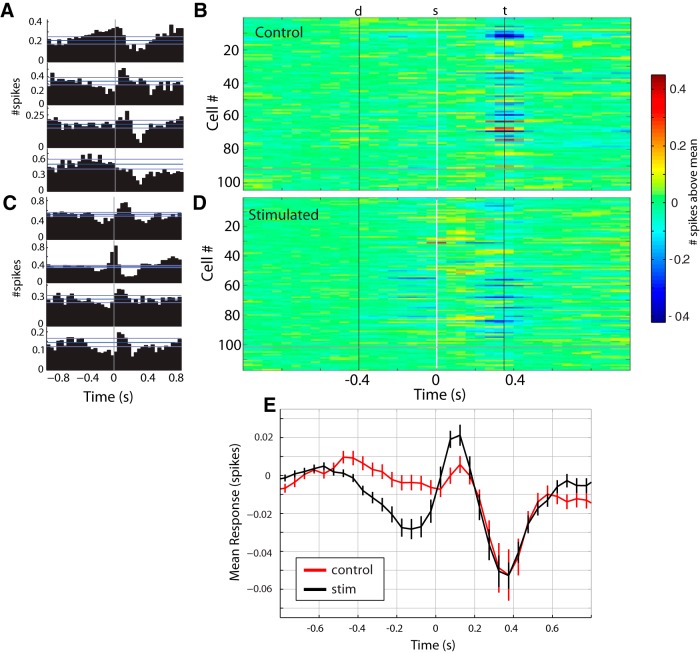
Neuronal responses during the aperture-discrimination task. ***A***, Four example PSTHs during the aperture-width discrimination task described in Fig. 5*A*, from control animals that were not stimulated. Bin widths are 50 ms. Reference lines show mean ± SD during the baseline period (the period before –400 ms). Time 0 is the time of stimulus onset, when the whiskers contact the aperture. ***B***, Set of all 104 PSTHs from two control animals. d, door opens to start trial; s, stimulus onset (whiskers contact aperture); t, tone sounds indicating rat has reached end of stimulus chamber. Spike counts in ***B*** are calculated by subtracting the mean spike count during the baseline period. PSTHs below the gray line are those for which there was no significant response. ***C***, ***D***, Same as ***A*** and ***B*** but for the stimulated animals (*n* = 117 neurons). ***E***, Mean ± SEM PSTH for all neurons for control (red) and stimulated (black) groups.

We next examined neuronal activity in S1 during performance of the aperture-width discrimination task in the stimulated and control groups. It is known that S1 neurons in animals trained to use S1 in an ICMS-based IR discrimination task maintain their ability to respond to whisker deflection outside of a task context (Thomson et al. 2013; Hartmann et al. 2016). Here, we recorded from populations of S1 neurons as two stimulated and two control animals performed the two-width aperture-width discrimination task. Fig. 6*A*, *B* shows sample PSTHs and the set of all PSTHs from all 104 S1 single units from the control animals, respectively. Fig. 6*C*, *D* shows the same for the 117 S1 units in the stimulated animals.

Overall 87% of neurons in the control group and 86% of those in the stimulated group displayed a significant response at some point in the trial. However, there were some quantitative differences in the two groups of animals. As has been reported extensively before, S1 neuronal activity during the aperture-width discrimination task can be analyzed in different time epochs around the instant of whisker contact with the port edges (Krupa et al. 2004; Pais-Vieira et al. 2013; Thomson et al. 2014). For instance, anticipatory activity in S1 has been commonly observed during the 400 ms that precedes the contact between the whiskers and the aperture (Krupa et al. 2004; Wiest et al. 2010; Pais-Vieira et al. 2013). We observed such anticipatory activity here as well, and the proportion of neurons showing such responses was larger in the stimulated group (0.71) than the control group (0.51; *p* = 0.009; chi-squared test for proportions). Further, the proportion of neurons responding to whisker deflections in the stimulus epoch (the period 350 ms after whisker contact with the aperture) was larger in the stimulated group than the control group (0.84 vs. 0.73), although this difference was not statistically significant (*p* = 0.053; chi-squared test).

## Discussion

We demonstrated that primary sensory areas in adult rats exhibit a noteworthy ability to absorb novel sources of information without compromising preexisting sensory function. It seems that primary cortical sensory areas in mammals can rapidly integrate two independent sensory streams while exhibiting very little large-scale interference. As was pointed out previously (Hartmann et al. 2016), the fact that the novel information source was IR light was arbitrary; other information sources, such as X-rays or microwave radiation, could be used if an appropriate portable sensor were available.

### Comparative analysis: accelerated learning with area-specific pretraining

This study is a first step in a comparative neuronal, behavioral, and functional analysis of the absorption of novel sources of sensory information in multiple primary sensory areas (Yang and Zador, 2012). Based on the anatomic and functional similarities in different sensory areas (Miller, 2016), and the fact that diverse areas can so readily acquire new functions in juveniles (Sur et al. 1988), we expected both V1 and S1 to exhibit the same properties when presented with IR information.

This is the result we observed, as rats implanted with S1 and V1 prosthetic devices both learned at the same rate (Fig. 2*F*). However, when pretrained on a structurally similar visual discrimination task, significant differences emerged between the two populations: the V1-implanted animals learned the task significantly faster than those implanted in S1 (Fig. 2). Typically, after pretraining on a visual discrimination task, V1-implanted rats learned the corresponding IR discrimination task during their first day of training, several days and hundreds of trials ahead of their S1-implanted counterparts.

When this visual pretraining was removed, this difference in learning rates between S1 and V1 learning rates disappeared (Fig. 2*F*), suggesting that there is no intrinsic difference in learning rates between the two populations. Cumulatively, these data support our initial hypothesis that there is an equipotentiality of plasticity across adult primary sensory areas: they are equally capable of absorbing a new source of distal sensory information and using it to perform a sensory discrimination task.

However, when pretrained on a structurally similar task that relies on a particular primary cortical area, there was a much more rapid transfer of learning when the new information was projected to that same primary cortical area (Fig. 2*A*, *E*). This suggests there exists a form of metaplasticity, in which the V1-implanted animals have learned to learn the task, transferring their earlier training to the new task. Such phenomena have been observed in the whisker system (Harris et al. 1999) and in cross-modal learning transfer (Over and Mackintosh, 1969). We do not yet know the underlying reason for this rapid transfer of learning, but speculate that during the visual task attention is already directed to V1, so the process of extracting and using the information from the prosthesis is facilitated in the V1-implanted animals. One way to test this hypothesis would be to pretrain the animals on a structurally similar tactile discrimination task and see whether the S1-implanted animals learn the IR-discrimination task faster than the V1-implanted animals.

Originally, it seemed a natural hypothesis that the V1-implanted animals learned faster because V1 is simply a more natural home for IR information: IR light is a distal electromagnetic cue, which V1 already processes, so there is no need to adapt new behavioral strategies for processing information as there is in S1. However, the fact that S1 and V1 learning rates converge when the animals are not pretrained on a visual discrimination task suggests there is no such intrinsic preference for IR information in V1 compared with S1.

### Multimodal integration within and across primary sensory areas

With the visual-IR integration task used in this study, information from both the visual and IR modalities had to be integrated and compared for rats to successfully complete a trial. If the animal solely focused on one type of information, they would incorrectly approach a distractor port (Fig. 3*A*). This is typically what happened at first, but within four sessions, subjects learned to approach the correct port (Fig. 3*B*). In the future, we will merge such multimodal stimuli in different combinations and intensities to more thoroughly quantify how animals integrate IR and visual information, in particular to measure how closely they approach the theoretical Bayesian optimum, as when variability is added to one of the cues, making it less reliable than the other (Deneve and Pouget, 2004; Dadarlat et al. 2015).

Surprisingly, there was no significant difference in performance in the visual-IR integration task when the IR information was projected to S1 or V1 (Fig. 3*C*). We had expected the S1-implanted animals to learn faster because there would be no intra-area sensory interference between the visual and IR modalities. Instead, our findings suggest that it may not matter whether the two information sources are projected to the same or different cortical areas.

The limits of such multimodal sensory augmentation are unknown. How many different types of information can be projected to one sensory area before the animal becomes confused? This question could have direct clinical relevance: in designing closed-loop feedback systems for artificial limbs, researchers aim to provide subjects with multiple types of sensory information, such as temperature, pressure, and proprioception (Donati et al. 2016). Hence, it will be important to determine the practical limits of augmented brain function.

### Conservation of native sensory function

It has been shown that when IR information is projected to the whisker region of S1, S1 neurons still show robust response to whisker deflections (Thomson et al. 2013; Hartmann et al. 2016). However, it was not known whether this cortical sensory neuroprosthesis would impair the native sensory function of the target cortical area. Would S1 become effectively hijacked by ICMS, and less capable of processing tactile information (Griffin et al. 2011)? There is evidence from monkeys that learning to perform ICMS-based threshold detection tasks in V1 impairs performance on visual detection tasks (Ni and Maunsell, 2010). Contrary to such findings, when we trained rats with the ICMS-based prosthesis on a whisker-based tactile discrimination task, we found no decline in the animals’ tactile discrimination (Fig. 5). Therefore, our results suggest that projecting novel sensory information to a primary sensory cortical area does not necessarily compromise the ability of the animal to use that area for sensory discrimination.

There are multiple differences between our paradigm and those in the previous study that could explain the different results. The previous research used a detection task in fixated, head-fixed animals and purposely limited the cue to a small region of visual space (Ni and Maunsell, 2010). By comparison, we used a discrimination task in which freely moving animals swept multiple whiskers across the target stimulus: this stimulus is known to activate a broadly distributed population of neurons across the trigeminal system (Krupa et al. 2004; Ferezou et al. 2006; Pais-Vieira et al. 2013, 2015). Similarly, the IR prosthesis was used by freely moving animals, with information distributed to four locations, in animals trained to forage for IR information in their environment over extended time scales, rather than a single 250-ms microstimulation train localized to a single location, as in the previous study (Ni and Maunsell, 2010). Finally, detection and discrimination tasks are quite different tasks, likely recruiting different underlying brain states optimized to their particular goal (Sherman, 2001). For instance, when performing a detection task, neurons in the cortex and thalamus may tend to fire in bursts, which is optimal for indicating that an event has occurred, but not for tracking its fine-grained features. When in discrimination mode, thalamic neurons exhibit more tonic background firing rates, and may track features of the environment at a finer grain and respond with lower magnitudes than in detection mode (Fanselow and Nicolelis, 1999; Adibi and Arabzadeh, 2011; Ollerenshaw et al. 2014).

Overall, although ICMS use may selectively increase localized detection thresholds (Ni and Maunsell, 2010), this does not mean that it harms performance in discrimination tasks in freely moving animals actively extracting sensory information from their environment.

In conclusion, our findings demonstrate that the mammalian brain remains extremely sensitive to the statistical structure of the inputs it receives throughout adulthood. This has long been clear from studies of newborns (Frost and Metin, 1985; Sur et al. 1988) and deafferented adults (Kaas et al. 1983; Kossut et al. 1988; Nicolelis et al. 1993). The current study shows that it is possible to superimpose, without any deafferentation, multiple streams of information simultaneously onto the same primary cortical area, whether it be S1 or V1. In this context, the present study strongly suggests that the upper limits of plasticity of a primary sensory cortical area, as well as its ability to adaptively absorb and use multiple sources of information, are much greater than previously anticipated. This is auspicious for the development of sensory cortical prosthetic systems, in particular those that might require the use of sensory substitution systems, such as those in which visual information is projected to the somatosensory system (Bach-y-Rita et al. 1969).

## Synthesis

Reviewing Editor: Leonard Maler, University of Ottawa Decisions are customarily a result of the Reviewing Editor and the peer reviewers coming together and discussing their recommendations until a consensus is reached. When revisions are invited, a fact-based synthesis statement explaining their decision and outlining what is needed to prepare a revision will be listed below. The following reviewer(s) agreed to reveal their identity: Ron Frostig, Beata Jarosiewicz

Editor's comments.

The reviewers and I all agree that this Ms advances the field at both basic science level and with the possibility of a new approach to neural prosthetic engineering.

Although both reviewers (and I) found that the Ms is certainly deserving of publication in eNeuro, they also found numerous points that needed attention.

I have included the detailed comments made by both authors do assist you in making revisions. Please pay special attention to the “Statistical Analysis” comments.

When you re-submit this Ms, be sure to indicate precisely how you addressed each of the reviewers detailed comments.

Reviewer #1

The authors keep telling the reader that the visual cortex results are similar to the somatosensory ones, 'but', or 'however', or 'there is a wrinkle', in that that pre-trained animals are learning significantly faster. Unless the authors have evidence that pre-training with whiskers is not improving their learning, the reader is confused on why this is a 'wrinkle'. I think that in the abstract, results and discussion the authors should start with the fact that the results are similar in both cortices (V1 and S1) and, in addition, pre-training improves learning and so far, this has been demonstrated only in visual cortex. 

Can the authors explain why there is a need to stimulate above the neuronal threshold (and raising twice the saturating current)? Lines 239-240 

It is rather disappointing that such an advanced lab that applies top sophisticated stimulating, behavioral, electrophysiological, and analytical techniques maps the whiskers' receptive fields using hand-held probe and listening to an audio output for the strongest responses (lines 244-246). I think that the authors should therefore tone-down their claims about receptive fields. Indeed, the locations of receptive fields was the only clear difference between V1 and S1, which is rather surprising given all other similarities.

It will strengthen the manuscript if the authors can site literature on how long does it take animals to learn a new discrimination task once they were pre-trained on another - especially if such duration is similar to their results. 

As cited in the manuscript it was already demonstrated in the past that adding an IR to S1 does not reduce or antagonizes processing of whiskers information. The authors should do a better job in explaining the rationale for testing this issue again. 

Can the authors provide a histological verification for the location of the stimulating electrodes? 

Figure 5: The authors should explain better panels D, E, F, G. 

Figure 6: The legend is quite short and there are several questions about this figure that are not explained anywhere: panel B, C and D where is the stimulus artifact? A color bar is missing; the stimulated panel (D) seem to contain neurons with stronger firing at all times compared to controls (B) but this does not seem to be reflected in E; it is not clear where is pre-touch excitation; is the 0 time the time of touch? 

Minor: 

Line 87 - missing information 

Line 103: remove the word 'for' 

Lines 121-122: 'left source to left cortical hemisphere': shouldn't it be the opposite (i.e., left source to right hemisphere')? 

Line 213: Figure 1D should be 1C 

Line 229: There is no Fig. 1E 

Line 577: Did you mean Figure 5? 

Line 703: Missing information 

Reviewer #2

Statistical analysis

Very little information was provided about the (bizarrely random) choice of N for each experiment and condition. It's difficult to evaluate whether the results support the conclusions without understanding the rationale for having, e.g.. 6 animals in the V1 implant condition but 16 (or 15, depending on which section it's written in) in the S1 implant condition. How were these numbers decided on? If in advance, by a power analysis or other objective method, one would expect the same number of animals for each condition. Also, why are the numbers inconsistent throughout the text? How were animals selected for follow-up experiments? Was the decision made that data collection was done for a given experiment ad-hoc, or even based on the results obtained thus far? 

Also, statements were made about statistical results that implied a lack of understanding of the meaning of p-values and the limitations of frequentist statistics (e.g. “X was larger than Y, but not significantly so”; “X and Y were statistically indistinguishable”; etc.) 

It is difficult to follow the logic of and motivation for the many experiments and conditions described in the Results section. Instead of “Next, we tested whether...” (the temporal sequence in which the analyses were performed are of little relevance to the reader), it would be useful to explain the actual motivation for each experiment (e.g. how the previously discussed experiment's results led to the next question, etc.). The Discussion section did a better job of explaining, one by one, the motivations for each of the experiments, but at that point, it's too much to ask the reader to re-read the Results within that context. In fact, I would recommend that the contents of the Discussion section be moved into Results, replacing it entirely, and just adding in the statistics where appropriate. (With the exception of the last section of the Discussion, which contained overarching and general discussion points that do actually belong in the Discussion.) 

- The seemingly random, sometimes conflicting, and sometimes unspecified number of animals per experiment was worrisome (for example, p. 6: “The majority of animals were initially trained on a visual discrimination task.”) Why the majority instead of all? Why not list the actual number? Why was the number of S1-stimulated animals listed as N=15 on p. 15 (line 300), but as N=16 in the corresponding figure legend (Fig. 2B)? What was the rationale for having 16 (or 15?) animals in the S1 implant condition but only 6 in the V1 implant condition? Another example is on p. 9, introducing the Aperture-width discrimination experiment: “We trained rats to discriminate the width of a tunnel they ran through, using only their facial whiskers.” Was this the same batch of rats as one of the other experiments, or a new batch of rats? How many rats were used? And another example on p. 10: “we then switched some rats to a version with either 12 or 14 widths...” Why some rats instead of all? How many of them, and why those particular rats? 

- Was the number of animals for each experiment selected in advance based on some objective criterion (like a power analysis)? The number of animals in each experiment and condition should be listed in the relevant section of Methods, and the rationale for each choice of animal count should be explained. 

- Was the training of S1 and V1-stimulted rats temporally interleaved? (If not, it might be difficult to know whether other unintentional differences were introduced between groups, such as gradual changes over time in the training protocol, experience of the surgeon doing the electrode placement, experience of the handlers and trainers, etc.) Were other potentially confounding differences between groups of rats controlled, such as the litters or shipments from which they were taken? 

- Many of the statements about results of statistical analyses took the form “x was larger than y, but not significantly so.” (E.g. bottom of p. 23; top of p. 24; top of p. 25.) If a difference is not statistically significant, then there is no evidence that a difference truly exists; thus, such statements should not be made. 

- How precisely was the information from the IR sensors mapped to the stimulating electrodes? Only a very gross-scale explanation is given, but even that is explained in 2 opposite ways in 2 different places. P. 7: “The information was projected in a topographically natural way, for instance, information from the left sensors was projected to the left cortical hemispheres (Hartmann et al., 2016).” But p. 15 states the opposite: “We ... projected information from IR sensors to the cortex in a topographically natural manner, such that the information from the left IR environment was projected to the V1 of the right hemisphere, and so on.” (Also, what does “and so on” mean here?) 

- P. 7: in the 'IR+vis' trials, IR light (via ICMS) was “followed by visible light, to help animals associate ICMS with reinforced visual light.” What was the timing of the IR-visible light presentation? 

- p. 9: Is IR level the same thing as IR intensity and IR sensor voltage? (If so, it would help reduce confusion to choose just one term for each concept.) 

- p. 10: “Locations of electrodes were verified histologically ...”: it would be helpful for the reader to see the histology results, and a summary plot of electrode placement (on a common brain, perhaps) for all animals with V1 electrodes and all animals with S1 electrodes. Was there truly a sharp distinction between S1 and V1-implanted rats' performance on the initial task, or was there possibly a more gradual relationship between how posterior the stimulating electrodes were and the speed with which the task was learned? 

- p. 16, bottom paragraph: “Next, we tested whether V1-stimulated animals learned to discriminate IR sources faster because they were pre-trained in a visual discrimination task that was structurally identical to the subsequent IR discrimination task.” I found this section very confusing, both in terms of the question being addressed, and the methods used to address it. Weren't both the V1 and S1-stimulated animals pre-trained in exactly the same visual discrimination task that was structurally identical to the subsequent IR discrimimation task? (I had assumed that the only difference in the between V1 and S1-stimulated animals had been the electrode placement.) The results of this experiment are difficult to follow, too: “The naïve V1-implanted animals took 4.0{plus minus}0.57 days to learn the IR discrimination task, which was statistically indistinguishable from the S1-implanted animals pre-trained on a visual task (p=0.85; two-tailed t-test). It was also the same as three S1-implanted animals that were not pre-trained on a visual task (4.33{plus minus}0.33 days; p=0.64, 2-sided t-test).” Does this mean that pre-training on a visual task *does not* influence speed of learning when stimulation is applied to S1, but pre-training on a visual task *does* influence speed of learning when stimulation is applied to V1? Can you break down your rationale for the hypothesis being tested here, and the implications of the results? (Also, “not significantly different” using frequentist statistics does NOT imply “statistically indistinguishable”; it just means that no conclusions can be drawn about the null hypothesis.) 

- bottom of p. 26: “We had expected the S1-implanted animals to learn faster because there would be no intra-area sensory interference between the visual and IR modalities. Instead, our findings suggest that it may not matter whether the two information sources are projected to the same or different cortical areas.” A reader might have expected the opposite, that V1-implanted animals might learn the IR task faster, because V1 natively processes information that has properties similar to IR light, unlike S1; i.e. it emanates from a source distal to the body, subtending a larger angle as you move closer, having the sensory-motor contingencies of a visual input source, etc.). Maybe both factors were at play, and simply balanced each other out, rather than it truly not mattering whether the 2 information sources are projected to the same or different cortical areas? 

- The writing needs careful editing (it is full of typos, grammar errors, and incoherent / incomplete sentences). For example: 

p. 11: “Such pulses were delivered at frequencies that as a monotonically nondecreasing function of IR level detected, from zero to 400 Hz (Figure 1E).” 

p. 6: “On the inner surface of each port a 96 visible LED and an infrared LED (Opto Semiconductors Inc., 940 nm peak emittance (range of 97 non-zero emission was between 825 and 1000 nm)).” 

p. 12 vs. 13: Inconsistent use of variable names in equations: lower-case f was the microstimulation frequency on p. 12, and Cohen's statistic on p. 13. 

p. 10: description of surgery flips back and forth between past and present tense several times. 

p. 10: “The cortical electrode configuration is illustrated in Figure 1D.” I assume you mean Figure 1C? Same thing at the end of the paragraph. (Also, it would be very helpful if you could label your figures with figure numbers.) 

- Given that this is a scientific report (and not a review or opinion piece), it might be appropriate to tone down the many hyperbolic and subjective modifiers throughout the text. E.g. In first paragraph of intro: “A fundamental goal in neuroscience...”, “This central question”...; In the Discussion: “... exhibit a remarkable ability to absorb completely new sources of information...”; “...surprisingly fine-grained resolution...”; “merely an academic enterprise” (this phrase might even be perceived as offensive to people with careers in academia); “the mammalian brain remains extremely sensitive...”; “Our study adds a completely new dimension to such work...”; “This is extremely auspicious...” I recommend removing these dramatizations and letting the science speak for itself. 
